# Effects of physical training on the metabolic profile of rats exposed to chronic restraint stress

**DOI:** 10.1016/j.clinsp.2024.100411

**Published:** 2024-06-19

**Authors:** Carlos H.O. Reis, Sabriny G. Manzolli, Leonardo dos Santos, Ariana A. Silva, Ana Paula Lima-Leopoldo, André S. Leopoldo, Danilo S. Bocalini

**Affiliations:** a Experimental Physiology and Biochemistry Laboratory, Physical Education and Sports Center of the Universidade Federal do Espirito Santo – Campus Goiabeiras, Vitória, ES, Brazil; b Cardiac Electromechanics Laboratory, Postgraduation Program in Physiological Sciences, Health Sciences Center, Universidade Federal do Espirito Santo - Campus Maruipe, Vitoria, ES, Brazil

**Keywords:** Stress, Glucose Tolerance, Physical exercise and fitness

## Abstract

**Introduction:**

Despite strong evidences supporting the protective role of exercise against stress-induced repercussions, the literature remains inconclusive regarding metabolic aspects. Therefore, this study aimed to evaluate the effect of Physical Training (PT) by swimming on the metabolic parameters of rats subjected to restraint stress.

**Methods:**

Wistar rats (n = 40) were divided into four groups: Control (C), Trained (T), Stressed (S), and Trained/Stressed (TS). The restraint stress protocol involved confining the animals in PVC pipes for 60 minutes/day for 12 weeks. Concurrently, the swimming PT protocol was performed without additional load in entailed sessions of 60 minutes conducted five days a week for the same duration. The following parameters were analyzed: fitness progression assessed by the physical capacity test, body mass, serum level of glucose, triglyceride, cholesterol and corticosterone, as well as glycemic tolerance test, evaluated after glucose administration (2 g/kg, i.p.).

**Results:**

Trained groups (T and TS) exhibited enhanced physical capacity (169 ± 21 and 162 ± 22% increase, respectively) compared to untrained groups (C: 9 ± 5 and S: 11 ± 13% increase). Corticosterone levels were significantly higher in the S group (335 ± 9 nmoL/L) compared to C (141 ± 3 nmoL/L), T (174 ± 3 nmoL/L) and TS (231 ± 7 nmoL/L), which did not differ from each other. There were no significant changes in serum glucose, cholesterol, and triglyceride levels among the groups. However, the glycemic curve after glucose loading revealed increased glycemia in the S group (area under curve 913 ± 30 AU) but the TS group exhibited values (673 ± 12 AU) similar to the groups C (644 ± 10 AU) and T (649 ± 9 AU).

**Conclusion:**

Swimming-based training attenuated stress-induced corticosterone release and prevented glucose intolerance in rats, reinforcing the importance of exercise as a potential strategy to mitigate the pathophysiological effects of stress.

## Highlights

• Swimming training significantly prevented stress-induced corticosterone increase in rats.
• Serum glucose, cholesterol and triglyceride were unchanged by chronic stress or exercise.
• Chronic stress protocol induced glucose intolerance in the rats.
• The training preserved the normal glycemic response after glucose loading in stressed rats.

## Introduction

Stress is considered a state of homeostasis disruption characterized by specific and generalized adaptive responses to a perceived threat, challenge, or demand caused by psychological, environmental or physiological agents.^1^ It can be categorized as either acute or chronic, both eliciting Sympathetic Nervous System (SNS) hyperactivity, catecholamine release, and dysregulation of the Hypothalamic-Pituitary-Adrenal (HPA) axis, resulting in elevated production of glucocorticoids ‒ cortisol in humans and corticosterone in rodents,^2^ recognized as primary stress indicators.^3^

While short-term stress responses help individuals cope with immediate challenges, chronic or severe stress can adversely affect both physical and mental health, contributing to the development of pathological conditions, particularly cardiovascular diseases.^4^ Additionally, the increased SNS and HPA activities are associated not only with increased heart rate and blood pressure, and suppression of the immune system but also with insulin resistance^7^ and increased serum levels of glucose and lipids.^5^

Several strategies have been explored to combat stress-related complications.^8^^,^^9^ Among these, physical exercise stands out as a clinically important tool in the control of stress symptoms.^10^ Increased physical activity and reduced sedentary behavior are particularly crucial strategies in preventing, managing, and treating various stages of functional impairment caused by chronic stress.^8^^,^^11^^,^^12^

Actually, a Silva et al.^6^ study conducted on rats exposed to chronic stress and physical training showed that adaptations induced by exercise attenuate stress-induced hypertension in rats but not the impairments on the myocardial mechanics. The significance of physical exercise is further evidenced by previous studies^8^^,^^11^ that have demonstrated positive outcomes in clinical indicators of stress. Furthermore, physical exercise had been considered such effective strategy to reduce blood pressure,^13^ improved blood glucose control,^14^ increased insulin sensitivity,^15^ enhanced immune response,^16^ and improved functional capacity.^17^

However, if considering the research with basic approaches, the knowledge of physical exercise interventions by swimming to treat and in preventing metabolic outcomes associated with stress is still unclear. To understand the effects of stress in a more invasive way, protocols used in animal models are developed to advance the discovery of the mechanisms involved in stress, as well as the physiological and morphological repercussions^18^ and understanding the interaction between various stimuli associated with physical activity.^19^ Thus, this study aimed to evaluate the effect of physical training by swimming on the metabolic parameters of rats subjected to restraint stress.

## Material and methods

### Animals and experimental groups

Twenty-nine male young Wistar rats (10‒12 weeks-old), weighing 200‒250g, were sourced from the Animal Facility at the Federal University of Espirito Santo for inclusion in this study. The animals were housed individually in cages, provided with “*ad libitum*” water and Nuvital CR-1 rat feed, in a controlled environment maintained at the temperature of approximately 22°C, humidity at 54%, and a 12-hour light/dark cycle. All protocols were in accordance with the recommendations of the National Council for Animal Experimentation. This study was previously approved by the Institutional Ethics Committee on Animal Use (number 21/2021).

The animals were randomly divided into four experimental groups as follows: Control (C; n = 10), consisting of animals that remained at rest for twelve weeks; Trained (T; n = 10), with animals that underwent twelve weeks of swimming training; Stressed (S; n = 10), animals that remained at rest and submitted to restraint stress for twelve weeks; and Trained/Stressed (TS; n = 10), comprising animals that underwent twelve weeks of swimming training while simultaneously being subjected to restraint stress.

### Physical exercise protocol

The swimming exercise protocol follows the procedures previously established by this group.^20^ The training program began with a 6-day adaptation phase, during which animals swam for 10 minutes on the first day, with each subsequent session adding ten minutes until completing 60 minutes per session. Then, a training phase was applied and maintained (60 minutes per session, five days a week) for 12 weeks. The pool water was constantly agitated using a pump to prevent the animals from diving or floating during the sessions, and a thermostat-controlled electric heating system maintained the water temperature between 32‒34°C, according to recommendations for rodent exercise regimes.^21^

### Chronic stress protocol

Chronic stress was attained using immobilization individually in a Polyvinyl chloride (PVC) cylinder. Restraint stress is a widely utilized model of emotional stress in animal studies.^12^^,^^22^ In the present study, animals were individually restrained in an opaque PVC cylinder (brown), measuring 20 cm in length and 6 cm in diameter, with closed ends and perforations for air circulation and maintenance of the ambient temperature at 25°C. This restraint lasted for 60 minutes per day, five days a week, over 12 weeks, according to established protocol.^12^^,^^22^ This confinement severely restricts the animal movement, allowing only respiratory movements. In addition, the animal was kept away from contact with feces and with the tail raised, since these factors could intensify the stress stimulus beyond the intended level.

### Physical fitness assessment

The physical fitness and capacity were assessed in all experimental groups at the beginning and end of the protocols, using the maximal physical capacity test as previously established.^20^ Briefly, animals were weighed, and loads equivalent to 10% of their body mass were affixed to their tails to assess physical capacity. The maximum swimming duration until the animal reached complete exhaustion was recorded, with exhaustion defined as submersion for at least 10 seconds.^20^

### Blood biomarkers associated with stress

After undergoing a 15-hour fast, glucose, cholesterol and triglyceride concentrations were measured from blood samples collected from the caudal vein using the equipment Accuttrend (Roche Diagnostic Brazil Ltda., Brazil).

Subsequently, rats underwent a glucose tolerance test. Baseline blood samples were obtained from the tail, followed by glucose administration (2 g/kg, i.p.), as protocols were previously established.^23^^,^^24^ Blood samples were then collected at 30, 60, 120, 180 and 280 minutes post-glucose administration. Glucose levels were determined using the same equipment (Accuttrend, Roche Diagnostic Brazil Ltda., Brazil), and glucose tolerance was analyzed based on the area under the glycemic response curve.

### Assessment of stress intensity

After 48 hours of physical fitness assessment, all animals underwent a 12-hour fast and were anesthetized with pentobarbital (Fontoveter Animal Pharmaceuticals and Cosmetics Ltd, São Paulo, Brazil) at a dosage of 50 mg/kg and euthanized by decapitation. Blood samples were collected in heparinized tubes, centrifuged at 3000 *g* for 15 minutes at 4°C, and the serum was stored in an ultra-freezer at −80°C. Corticosterone levels were determined using a specific radioimmunoassay kit (Coat-A-Count Rat Corticosterone – Diagnostic Products Corporation, Los Angeles, USA), with a detection limit of approximately 16 nmoL/L.

### Statistical analyses

Data are presented as mean ± Standard Error of Mean (SEM). The D'Agostino-Pearson test was applied to analyze the normality distribution. For comparisons, the two-way ANOVA test was used, complemented with Bonferroni's or Dunn's post hoc tests, as necessary. All tests and graphics were performed using GraphPad Prism software (version 8.0, GraphPad Softwares Inc., San Diego, CA, USA). The significance level was set at 0.05.

## Results

**Fig. 1:**
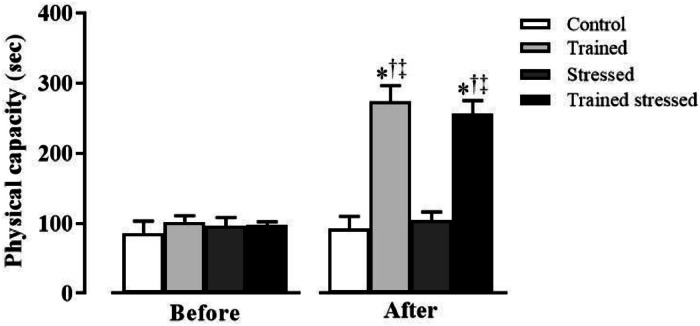
Values expressed as mean ± SEM of the physical capacity of animals in experimental groups before and after 12 weeks of intervention (* p < 0.05 vs. before; ^†^ p < 0.05 vs. C; ^‡^ p < 0.05 vs. E).

As shown in Fig. 1, there was no significant difference (p > 0.05) in the physical capacity of the groups before the protocols. However, after 12 weeks of training, the groups T (before: 102 ± 23 vs. after: 273 ± 61 seconds) and TS (before: 97 ± 14 vs. after: 256 ± 50 seconds) improved their physical capacity, without distinction between them. Moreover, the physical capacity of the untrained groups remained unchanged after this period (group C, before: 86 ± 45 vs. after: 92 ± 46 seconds and group S, before: 96 ± 33 vs. after: 104 ± 32 seconds). As a result, the training protocol was able to improve 169 ± 21% and 162 ± 22% of the physical capacity of groups T and TS, respectively.

**Fig. 2:**
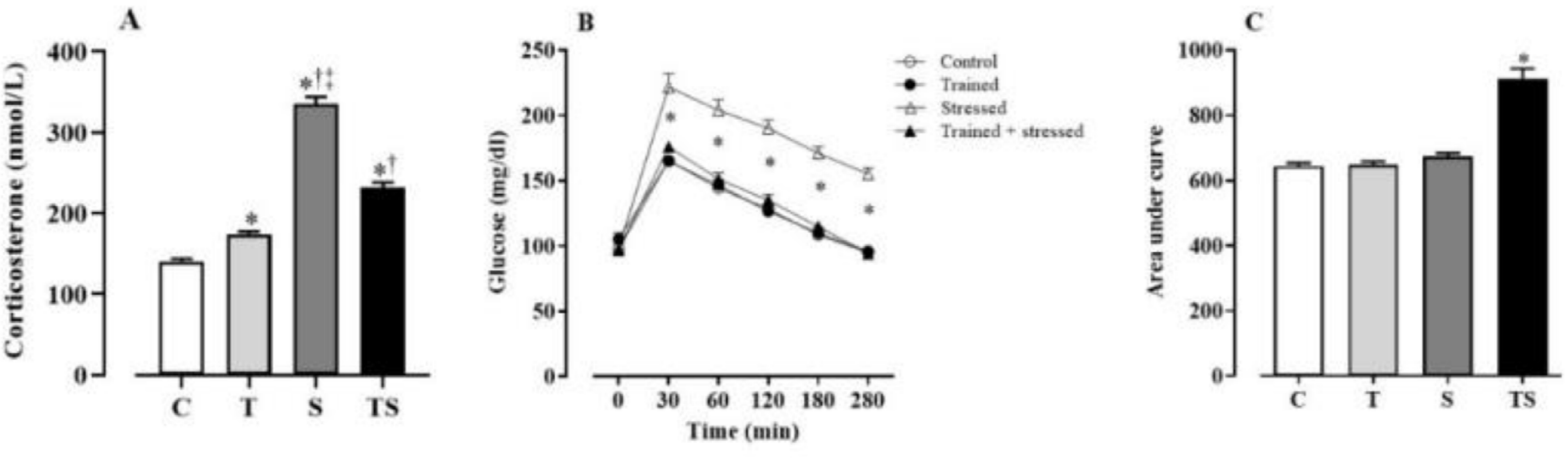
Values expressed as mean ± the SEM of corticosterone levels (Panel A), blood glucose curves after loading test (Panel B) and areas under the glycemic curves (Panel C) of groups C (control), T (trained), S (stressed), and TS (trained stressed) after 12 weeks of intervention (* p < 0.05 vs. C; ^†^ p < 0.05 vs. T; ^‡^ p < 0.05 vs. TS).

The corticosterone levels (Fig. 2A), as measured in the experimental groups 12 weeks after protocols, was significantly increased in the stressed animals (335 ± 9 nmoL/L) them C (141 ± 3 nmoL/L), T (174 ± 3 nmoL/L) and TS (231 ± 7 nmoL/L). However, the strength training slightly elevated corticosterone levels in nonstressed groups, this exercise protocol was able to partially prevent the corticosterone increase induced by the chronic stress. which also differed from each other. No effects on the basal levels (fasting) of serum glucose (C: 100 ± 4, T: 97 ± 3, S: 100 ± 4, TS: 97 ± 5 mg/dL), cholesterol (C: 161 ± 3, T: 156 ± 4, S: 178 ± 8, TS: 258 ± 2 mg/dL) and triglyceride (C: 123 ± 6, T: 123 ± 6, S: 130 ± 8, TS: 127 ± 9 mg/dL).

In terms of glycemic tolerance, as shown in Fig. 2B, the glucose levels in group S remained consistently higher (p < 0.05) following glucose administration compared to groups C, T, and TS, which showed no significant differences between them. When considering the overall blood glucose patterns throughout this protocol, the area under the curve values (Fig. 2C) for group S (913 ± 30 AU) was notably elevated compared to groups C (644 ± 10 AU), T (649 ± 9 AU), and TS (673 ± 12 AU), with no significant variances observed between the lattes three groups. Together, these findings clearly indicate impaired glycemic control in animals submitted to chronic stress protocol, which was significantly prevented by the strength-training program.

## Discussion

Physical inactivity is directly associated with various health risk factors, including stress,^26^ which can diminish life expectancy within the population.^25^ Conversely, regular physical exercise is recognized as an important protective measure against adverse effects of stress^6^ and numerous stress-associated chronic diseases such as cardiovascular diseases, diabetes, obesity^27^ and atherosclerosis.^28^

Considering the exercise protocol used in this study, this type of training has been widely employed to investigate the repercussions of physical exercise,^6^ proving effective in enhancing physical capacity,^29^ albeit potentially inducing acute stress-like effects.^6^ In the present study, even the physical capacity of animals subjected to stress was significantly improved by the training protocol, aligning with findings from previous reports.^6^^,^^30^ Additionally, serum corticosterone levels in animals exposed to chronic stress were not elevated when combined with physical exercise. Given that glucocorticoids are considered biological markers for chronic stress, elevated levels have been associated with an increased risk of developing conditions such as hypertension, atherosclerosis, and heart failure.^27^^,^^28^

No changes in glucose, cholesterol, and triglyceride levels were observed in this study, diverging from findings in other studies.^31^^,^^32^ It is postulated that duration, intensity, and volume of stress exposure may have influenced this outcome. Prior research^6^^,^^33^ indicated that different types of exercise confer benefits against stress-induced pathological changes. Thus, as demonstrated in the present study, physical exercise led to enhanced regulation of glycemic control.^34^^,^^35^ Although the precise mechanism underlying this improvement remains unexplored, increased glucose uptake by the muscle due to the increased amount and activity of the GLUT4 are suggested to play an important role in this response.^36^ Therefore, employing distinct pathways for GLUT4 translocation emerges as a principal mechanism for glucose uptake by skeletal muscle tissue. The energy discrepancy induced by muscle fiber contraction serves as the primary stimulus for AMP-Kinase (AMPKK), an enzyme that activates AMPK, subsequently facilitating the translocation of GLUT4 vesicles to the cell membrane.^36^

Furthermore, stress has been shown to compromise the insulin response.^37^ Since insulin plays a critical role in regulating blood glucose homeostasis, the development of insulin resistance is one of the main factors contributing to the onset of type II diabetes, a condition already correlated with stress.^38^ From this perspective, regular physical exercise has been recognized as an important strategy to improve insulin response.^32^ Although our the authors did not evaluate the insulin response in the present study, the authors believe that the preserved glycemic behavior of stressed and trained animals to glucose loading may be justified by an improved insulin response by some mechanisms previously documented in the literature.^14^^,^^35^ Nonetheless, further studies are needed to confirm this hypothesis.

As evidenced in this study, physical exercise led to a reduction in corticosterone levels^2^^,^^39^ and improved regulation of glycemic control.^34^ The decrease in corticosterone levels has been linked to a reduction in stress intensity, as observed in previous research;^40^^,^^41^ however, further investigations are warranted to fully understand this response. Elevated circulating glucocorticoid levels diminish the sensitivity of hepatic and peripheral tissues to insulin, resulting in increased hepatic glucose production and impaired glucose absorption by muscles and adipose tissue.^38^ In line with the authors's findings, plasma corticosterone levels were found to rise during and up to 1 hour after an episode of acute stress, coinciding with a return to baseline glycemic levels.^38^ As demonstrated in the present study, physical exercise attenuated corticosterone levels^2^^,^^39^ and improved regulation of glycemic control^34^ of the stressed rat. Actually, the decrease in corticosterone levels has been linked to a reduction in stress intensity, as observed in other studies;^40^^,^^41^ however, more investigations are warranted to clarify this response. Elevated circulating glucocorticoid levels diminish the sensitivity of hepatic and peripheral tissues to insulin, leading to an increase in hepatic glucose production and impaired absorption by muscles and adipose tissue.^38^ In line with the present findings, plasma corticosterone levels were found to increase during and up to 1 hour after an episode of acute stress, coinciding with a return to baseline glycemic levels.^38^

In general, the results of this study suggest that exercise effectively attenuated the stress effects on glucose control and corticosterone levels, thus serving as a protective strategy that influences the hypothalamic-pituitary-adrenal axis, and the secretion of Adrenocorticotropic Hormone (ACTH). This reduction in serum corticosterone levels aligns with similar results found in previous studies.^6^^,^^39^ For instance, by Droste et al.^42^ observed substantial changes in HPA axis regulation and body composition among exercised mice. Notably, their findings included the loss of adrenal asymmetry due to adrenocortical enlargement and sympathoadrenomedullary overstimulation, indicative of potential changes in the sympathoadrenomedullary system and hormonal secretion patterns in the HPA axis. The increased circulating corticosterone levels found in exercised animals compared to controls may be considered as anticipatory and adaptive responses aimed at supporting metabolism in the face of the demand of physical activity, without concomitant increases in ACTH release by the HPA axis.^43^ Therefore, sympathetic activity in the adrenal medulla serves as a positive modulator of adrenocortical sensitivity to ACTH. Thus, physical exercise appears to attenuate HPA axis activity in response to other stressors, providing an adaptive state with reduced ACTH stimulation,^43^ which may explain the observed reduction in plasma corticosterone levels.

## Conclusion

Animals subjected to restraint stress, even in the absence of basal glycemic alterations, exhibited impaired glycemic control, accompanied by glucose intolerance. However, this response was mitigated by physical training. Given that physical exercise is advocated by the World Health Organization and other health organizations for maintaining health, additional research is warranted to uncover its additional benefits in mitigating the adverse effects of chronic stress. Animals submitted to restraint stress, even in the absence of basal glycemic alterations, exhibited impaired glycemic control characterized by glucose intolerance. However, this alteration was mitigated by physical training. Given that physical exercise is advocated by the World Health Organization and other health organizations for maintaining health, additional research is warranted to uncover its additional benefits in mitigating the adverse effects of chronic stress.

## Funding

This research received no specific grant from any funding agency in the public, commercial, or not-for-profit sectors.

## CRediT authorship contribution statement

**Carlos H.O. Reis:** Visualization, Investigation, Writing – original draft. **Sabriny G. Manzolli:** Investigation. **Leonardo dos Santos:** . **Ariana A. Silva:** . **Ana Paula Lima-Leopoldo:** Visualization, Investigation, Writing – review & editing. **André S. Leopoldo:** Visualization, Investigation, Writing – review & editing. **Danilo S. Bocalini:** Visualization, Investigation, Writing – original draft, Writing – review & editing.

## Declaration of competing interest

The authors declare no conflicts of interest.
